# Steroidal Triterpenes: Design of Substrate-Based Inhibitors of Ergosterol and Sitosterol Synthesis

**DOI:** 10.3390/molecules14114690

**Published:** 2009-11-18

**Authors:** Jialin Liu, William David Nes

**Affiliations:** Department of Chemistry and Biochemistry, Texas Tech University, Lubbock, Texas, 79409, USA; E-Mail: jialin.liu@ttu.edu (J.L.)

**Keywords:** sterol C24-methyltransferase (24-SMT), transition state analogs, mechanism-based inactivator, ergosterol biosynthesis inhibitors, ergosterol, sitosterol, triterpenes

## Abstract

This article reviews the design and study, in our own laboratory and others, of new steroidal triterpenes with a modified lanosterol or cycloartenol frame. These compounds, along with a number of known analogs with the cholestane skeleton, have been evaluated as reversible or irreversible inhibitors of sterol C24-methyltransferase (SMT) from plants, fungi and protozoa. The SMT catalyzes the C24-methylation reaction involved with the introduction of the C24-methyl group of ergosterol and the C24-ethyl group of sitosterol, cholesterol surrogates that function as essential membrane inserts in many photosynthetic and non-photosynthetic eukaryotic organisms. Sterol side chains constructed with a nitrogen, sulfur, bromine or fluorine atom, altered to possess a methylene cyclopropane group, or elongated to include terminal double or triple bonds are shown to exhibit different *in vitro* activities toward the SMT which are mirrored in the inhibition potencies detected in the growth response of treated cultured human and plant cells or microbes. Several of the substrate-based analogs surveyed here appear to be taxa-specific compounds acting as mechanism-based inactivators of the SMT, a crucial enzyme not synthesized by animals. Possible mechanisms for the inactivation process and generation of novel products catalyzed by the variant SMTs are discussed.

## Introduction

**Figure 1:**
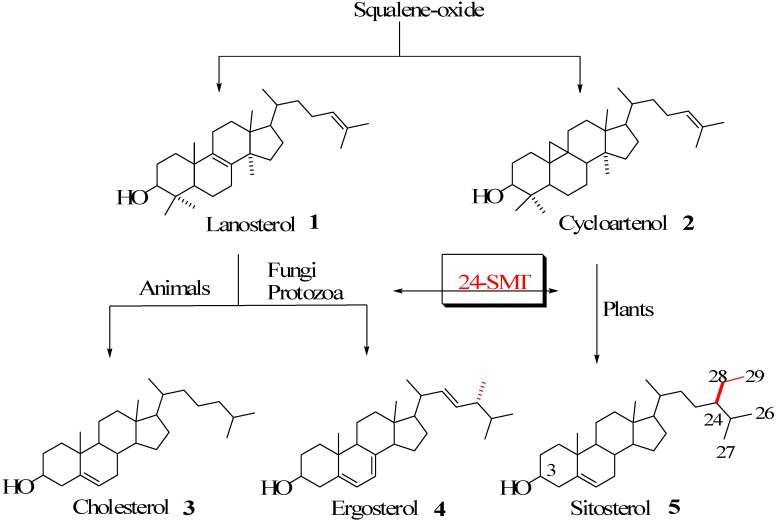
Biosynthesis of cholesterol and phytosterols.

Sterol metabolism is an extremely important area of biochemical differentiation between humans and their pathogenic microbes that might be exploited in the development of antifungal or antiparasitic drugs. In contrast to animals, both fungi and protozoa, in addition to plants, are able to synthesize the 24-alkyl side chains characteristic of the C_28_ and C_29_ membrane inserts ergosterol and sitosterol found in these organisms [1,2,3] (Figure 1).

The enzyme that catalyzes the conversion of Δ^24^-sterol acceptor molecules to 24-alkyl sterols is sterol C24-methyltransferase (24-SMT) [1]. This is often the rate-limiting step in phytosterol biosynthesis and sets the pattern for sterol specificity and diversity. Together these enzymes are responsible for the formation of more than 200 distinct 24-alkyl side chain structures [4], representing only a small fraction of the estimated natural variation seen in Nature [5]. 24-SMT monofunctional (C_1_-transfer activity) or bifunctional (C_1_ and C_2_-transfer activities) [3,6] enzymes are bound in the endoplasmic reticulum of the cell [7,8] and utilize two substrates, methyl donor AdoMet and Δ^24^-sterol acceptor, in the construction of the phytosterol side chain. The 24-SMT is not synthesized in animals, making it a novel target for rational drug design.

The crucial role of these enzymes in cell proliferation in eukaryotic microbes and plants has stimulated considerable interest in the enzymatic properties of these reactions in which the C24 alkylation of Δ^24^ -sterols is viewed as a nucleophilic attack of the Δ^24^ -π electrons on the *S*-methyl group of AdoMet [9]. Since the 24-alkyl sterol (ergosterol) to 24-desalkyl sterol (lanosterol/zymosterol) balance, representing the ratio of end product sterol to pathway intermediates, is considered to be a factor in ergosterol-forming disease processes [10,11,12,13,14,15], work in this laboratory and elsewhere has focused on the rational design and development of specific inhibitors of the 24-SMT. Notably, in-depth studies of the biochemical pathways of 24-methyl and 24-ethyl sterol formation in a wide range organisms have yielded important insights on the role of C24-methylation reactions in organisms that cause pathological states stimulating the development of new, selective model compounds that can disrupt phytosterol homeostasis. The structure-function aspects of sterol derivatives with modified side chains tailored to inhibit 24-SMT are reviewed in the present article.

## Steroidal Triterpenes

In the past 10 years, there have been several remarkable advances in the study of C_30_ compounds derived by the cyclization of 2,3-oxidosqualene using cloned enzymes that permit a distinction between them based on chemical analysis of their structure and stereoechemistry [16,17]. The conventional definition for triterpenes is any isoprenoid formed from six isoprene (C_5_-) units and having thirty carbon atoms. They can be acyclic, as in squalene, or cyclic, as in the tetracycles with a side chain typified in euphol, dammardienol or lanosterol and cycloartneol, respectively, or in the pentacycles, typified by the amyranes. 

**Figure 2:**
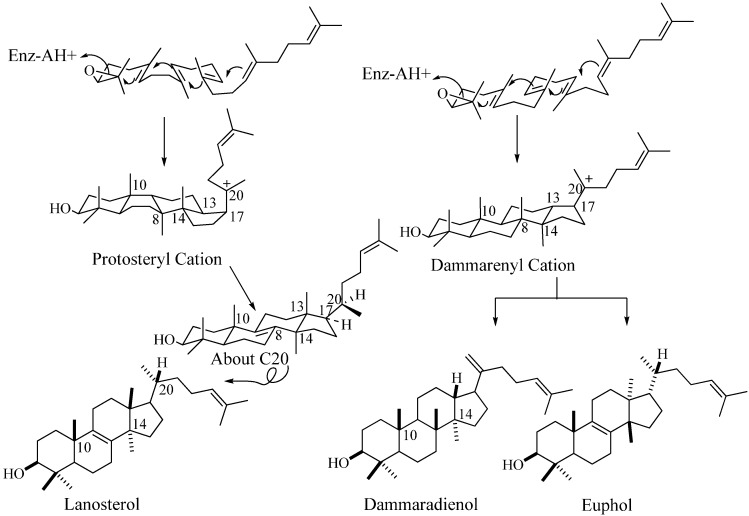
Cyclization of 2,3-oxidoqualene to sterols and triterpenoids.

In many descriptions of these compounds, the various structural classes are designated by the names of representative members. However, a steroidal triterpene can be categorized on their biogenic origin involving the squalene-oxide synthase (cyclase) [18]. For tetracycles derived by the cyclization of 2,3-oxidosqualene, the enzyme-bound stereochemistry of the protosteryl cation defines the intermediate structure. This intermediate will possess a nucleus conformation of chair-boat-chair-boat and C17α-unfolded side chain that converts to a final tetracycle frame characterized by the alternating all-trans-anti stereochemistry in the nucleus yielding a pseudo planar shape and a side chain that orients into a “right-handed” conformation due to the C20α-hydrogen configuration; they are true sterols possessing the functionally essential 20*R*-stereochemistry [1,18]. An alternate cyclization mechanism proceeds with the enzymatic formation of the dammarenyl (syn.= euphoid; triterpenoid) intermediate with the ring oriented in a chair-chair-chair-boat conformation converting to tetracyclic products of varied stereochemistry in the final structure (Figure 2). Lanosterol (formed in non-photosynthetic organisms) and its 9β,19-cyclo structural isomer cycloartenol (formed in photosynthetic organisms) convert to 24-alkyl sterols and only they are bound productively to the 24-SMT [1]. For these reasons, we will refer to the steroidal triterpenes as sterols and to 24-alkyl sterols as phytosterols. 

## Sterol C24-Methyltranserases

Over the past decade, knowledge of the biochemistry, molecular biology, and regulation of 24-SMT has increased significantly, as reflected in several recent reviews on the enzyme [2,19,20,21,22,23,24]. The fungal SMT gene from *Saccharomyces cerevisiae* (Sc), designated *ERG*6 and the best studied for this class of catalyst, encodes the Erg6p with a substrate preference for zymosterol (EC. 2.1.1.41) [25]. It consists of one extended exon (no introns) in the genomic organization distinct from plant SMTs that possess different levels of intron organization [26]. Three other classes of 24-SMTs have been characterized based on the C_1_ or C_2_-transfer activity and the corresponding substrate preference for the enzyme; plant SMT1; cycloartenol, (EC. 2.1.1.41), fungal SMT1, lanosterol (EC number not determined), plant SMT2 (24(28)-methylene lophenol (EC. 2.1.1.143)). As a class, the 24-SMT have been difficult to study because they are in low abundance in wild-type organisms and can be recalcitrant to crystallization for X-ray structure determination, thus providing limited opportunity to investigate the basic chemical reactions carried out by these enzymes using classic approaches. Alternatively, the cloning and over-expression of recombinant 24-SMT in *Escherichia coli* have now provided sufficient quantities of these interesting enzymes for structural and mechanistic investigations.

In the case of yeast 24-SMT, the full-length ScSMT cDNA of 1.52 Kb encodes a protein of 383 amino acids with a native molecular weight of approximately 172,000 Daltons and a single binding site for sterol and AdoMet [27,28]. The catalytic competence of this and several related 24-SMTs have been shown to be slow acting enzymes of *k*_cat_ 0.6 min^-1^ that recognize their optimal substrates with similar affinities (apparent *K*_m_ = ca. 35 µm and apparent *K*_d_ = ca. 4 µM) [1,28,29]. These membrane-associated 38-43 kDa proteins show 49 to 65% sequence identity between eukaryotic proteins. Consistent with their large overall sequence similarity, 24-SMTs possess four highly conserved regions, three of which contribute to the sterol binding pocket and one of them contains an AdoMet (SAM) binding pocket [29,30]. The sterol binding pocket incorporates α-helix turn motifs rich in aromatic amino acids proposed to be important in cation-π interactions involved with stabilization of the enzyme-bound intermediate [28]. In the absence of a three-dimensional structure of this class of catalyst, topology mapping by means of mechanism-based inactivators, photoaffinity probes and directed-mutagenesis have been undertaken by several groups to determine the general characteristics of the 24-SMT active sites. Kinetically, the mechanisms performed by the 24-SMT can differ; Erg6p operates a random bi bi such that either sterol or AdoMdet can bind first to the enzyme [25], whereas for the plant 24-SMTs the binding order is sequential whereby AdoMet binds first followed by the sterol binding to the enzyme [31].

**Figure 3:**
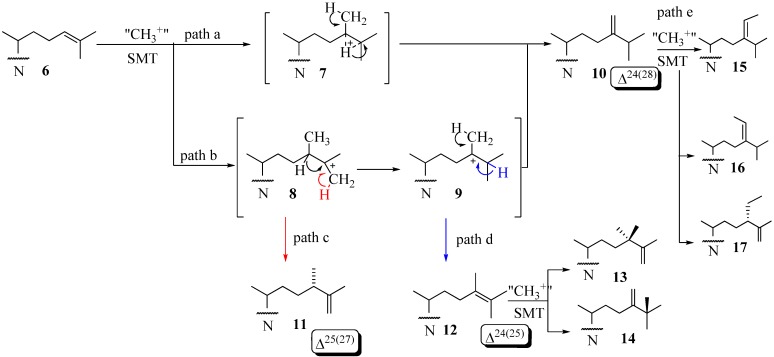
Various C-24-alkylation pathways.

The coupled methylation-deprotonation reaction catalyzed by 24-SMTs involves electrophilic alkylations of a remote double bond at C24 in the sterol side chain by a methyl cation originating with AdoMet, These enzymes act as a scaffold for hydride or methyl shifts as well as deprotonations that arise along the lateral sterol side chain while at the same time shielding cationic intermediates from premature quenching by enzyme nucleophiles or water long enough for C24-methylations and rearrangements to proceed along a controlled pathway. The majority of the characterized 24-SMTs are monofunctional, catalyzing a specific C_1_-transfer activity and possess very high substrate specificity yielding a single C24-methylated olefin. However, there are some enzymes that are bifunctional and recognize atypical substrates that convert to a variety of 24-alkyl(idene) products. For example, the *Trypanasoma brucei* TbSMT1 produce 24-methyl sterols that serve as substrates for 24-dimethyl sterols that contain a Δ^25(27)^ -bond [32]. *Glycine max* SMT1 can accept Δ^24(25)^ -24-methyl sterols and convert them to 24-dimethyl sterols with a C25- quaternary group in high yield or accept Δ^24(28)^ –sterols and convert them to a triplet of 24-ethyl(idene) products in low yield [31]. Structures **6,10** and **12** can serve as substrates that convert to C24-methyl(ene) or C24-ethyl(idene) sterol products via one of several paths [31,32,33,34,35,36] (Figure 3). Path d involving **12** is considered unusual in that novel methyl products are formed with a quaternary methyl group at C25 **14**. 

This enzymatic reaction can proceed by an intermediate possessing: (i) a bridged carbocation across the C24-C25 bond (path a) or (ii) a cationic site at C25 in the C24-alkylated sterol (path b) (Figure 3). Two explanations have evolved for the timing of the different C-methylation steps. One is the reaction is concerted [6]. This event has been confirmed for fungal ScSMT whereby the S_N_2 reaction proceeds by way of synchronous changes in bonding that occur with C24-methylation and C28-deprotonation that lead to the exocyclic Δ^24(28)^ -bond [25]. The other explanation is a nonconcerted process involving a series of conformationally rigid intermediates where topology is maintained between the initiation and terminating steps. C24- Alkylations that are concerted usually form a single Δ^24(28)^ -sterol product whereas those that are stepwise and stereoselective yield multiple products containing a Δ^24(28)^ – or Δ^25(27)^ -bond in the sterol side chain. The deprotonation reaction in olefin formation at C25(27) occurs exclusively from the *Z*-methyl group at C27 of the sterol Δ^24(25)^ -substrate as determined by incubations of [27-^13^C]labeled sterols to 24-SMT [6]. The 24-SMT can distinguish stereochemically modified (C20 *R* and *S*) or truncated Δ^24^-sterols with shortened side chains. For substrates of fungal or plant 24-SMT, the C20*R*-chirality and two methyl groups on the terminal double bond (isopropylidene moiety) were shown to be indispensable for productive binding and catalysis [37,38]. As discussed later in this review, a third (iii) catalytic transition may arise for the transalkylation of sterols in which the partitioning of select intermediates that fail to completely convert to product can inactivate the enzyme.

The nature and arrangement of the amino acid residues in the active site of individual 24-SMTs is crucial to the outcome of the reaction. In the case of single amino acid replacements directed at the ScSMT, amino acid substitution of 8 residues scattered throughout the primary structure have been shown to convert the activity of the fungal enzyme to plant-like activities [30]. On the other hand, comparative mutational analysis of similar residues in ScSMT that occur in the protozoan SMT1 [34] or in the soybean SMT1 failed to produce equivalent changes in product distribution [35]. Notably, a key tyrosine residue at position-81 in the conserved Erg6p Region I replaced with phenylalanine makes a great contribution to the acceleration of the C-24 methylation reaction and it can provide a gain-in-function toward the formation of the second C_1_-transfer activity [36]. While directed mutagenesis experiments indicated the high adaptability of these enzymes, in some cases the native substrates converted to unexpected outcomes showing possibilities for functional divergence [26].

## Molecular Parameters of Inhibitors Directed at the 24-SMT

The easiest inhibitors to design are substrate analogs, because they are likely to bind specifically to the active sites of the same enzymes as the substrates they resemble. For these inhibitors to be effective, they should bind much tighter than their normal substrates such that their delivery to the target enzyme can occur at concentrations that greatly exceed the affinity constant of the natural substrate. These substrate mimics should act as non-competitive inhibitors relative to the natural sterol acceptor and competitive inhibitors relative to AdoMet for tight binding to occur. The purpose of designing inhibitors targeted at the 24-SMT is two fold: (1) to obtain insight into the mechanism of the C24-methylation reactions, as well as to gain information on the active site topography, and (2) to develop leads to disrupt phytosterol homeostasis associated with disease states.

**Figure 4:**
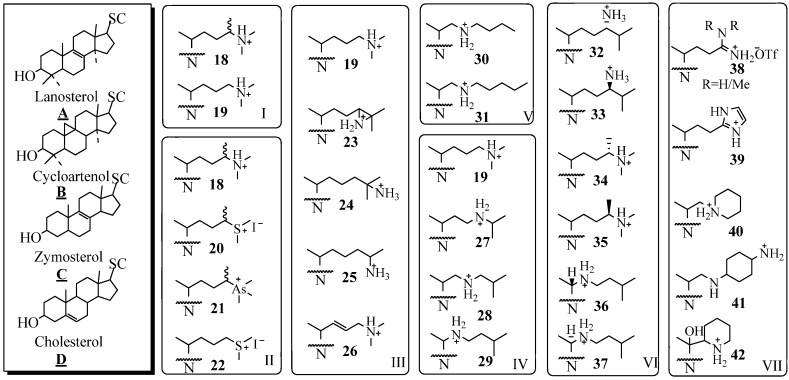
Sterol nuclei and modified side chains of test compounds; SC, side chain and N, sterol nucleus.

Investigations of the development of 24-SMT inhibitors began with the synthesis and biochemical evaluation of substrate-based inhibitors considered to be high energy intermediate analogs against the natural substrate (Figure 4). The first generation derivatives with a sterol nucleus and modified side chain were designed to mimic the C-24 methyl C-25 cationic intermediate **8**. These high energy intermediates (HIEs) analogs, also referred to as transition state (TS) analogs, were prepared by replacing C25 with an atom such as nitrogen that can acquire charge as a result of being protonated under physiological conditions. In seminal work, Benveniste and coworkers reported that 24(*R,S*)- methyl -25-azacycloartenol (Figure 4 **B18**) is a potent inhibitor of plant SMT1 (IC_50_ = 50 nM), [39]. Subsequently, 25-azacycloartenol **B19** and 25-azalanosterol **A19** were prepared and shown to be effectively equipotent inhibitors relative to the 24-methyl-25-aza sterols showing that the extra C24 methyl substituent is minimally important to activity [37,38,39,40,41,42].

**Figure 5:**
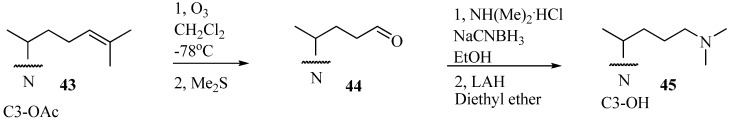
Preparative route to 25-azalanosterol **A45**.

The substrate specificity of the different 24-SMTs has been probed with a variety of substrate analogs of mixed nuclei and side chains, as summarized in Figure 4. By comparing the series **B19**, **A19**, **C19** and **D19** against different 24-SMTs, it was concluded that a correspondence exists between the dissociation constant (*K*_i_) and the nuclear structure. For example, the fungal ScSMT activity is inhibited according to the order: 25-azacholest-8-enol (*K*_i_ = 15 nM) > 25-azacholesterol (*K*_i_ = 25 nM) > 25-azalanosterol (*K*_i_ = 45 nM) > 25-azacycloartenol (*K*_i_ = 50 nM). The reverse order occurs for the four analogs tested with the plant SMTs from *Prototheca wickerhamii* and *Zea Mays* [38,40]. The effectiveness of substrate mimics toward their respective test 24-SMT agrees well with the substrate specificity of that enzyme, plant or fungal, toward cycloartenol **A6**, lanosterol **B6**, zymosterol **C6** and desmosterol **D6**. A typical preparative route for the synthesis of 25-azasterols, the most common analog tested with the 24-SMTs, is shown in Figure 5 [37,41].

By using a panel of substrate analogs shown in Figure 4 (Panels I to VII) with modified side chains but are otherwise similar to the natural substrates of side chain **6** or **10** and by assuming that the recognition of analog and corresponding substrate involve different interactions during the reaction, it has been possible to identify and evaluate at least two critical stereoelectronic elements of specificity related to the type and location of the functional group involved with inhibition [37,38,43,44,45,46,47,48,49,50,51,52]. Thus sterols containing a heteroatom at C24 or C25 of nitrogen, sulfur or arsenic are excellent inhibitors of 24-SMT [11,40,43,44,45,46]. The Panels I to III show analogs with modification in the isopropylidene region of the side chain that further reflect differences in atomic composition and valency. The enzyme recognizes all the test variants as reversible tight-binding analogs that inhibit the enzyme with a *K*_i_ in low nanomolar concentrations.

Structural modifications introduced along the lateral side chain were recognized by 24-SMT with decreased effectiveness (Panel IV). Thus, step-wise movement of the nitrogen atom from C25 toward C20 as in **19** to **27** to **28** to **29**, resulted in a marked decrease in *K*_i_ of the inhibitor by a factor 10. By increasing the substituent’s length toward the distal end of the side chain (Panel V) and keeping the nitrogen atom in a critical location at C22 resulted in a decrease in the dissociation constant of the inhibitor [15]. However, there was no essential requirement for an intact isopropyl group, since **25** was an effective inhibitor [36]. The steric requirement of C24-methylation inhibition was established by assay of the isomer pairs **32/33**, **34/35** and **36/37;** those that mimicked the natural intermediate having the C20*R* and 24β-methyl stereochemistry - **32**, **34**, and **36-** generated the most potent inhibition of 24-SMT. In Panel VII, extensive structural modifications of the sterol side chain are explored with respect to the chemistry of the *N*-substituent. Evaluation of the bulky amidine **38,** imidazole **39**, piperidine **40** and **42** or heterocycle with neighboring amines **41** or their congeners with 24-SMT showed similar inhibitory potency to the azasterols tested in Panel 4, i.e., inhibition of 24-SMT occurs in the nanomolar range of the drug tested. These results are consistent with the hypothesis that *N*-derivatives mimic the C25 carbocationic intermediates occurring in the C24-methylation step of the reaction mechanism. Most of the compounds known in the literature as inhibitors of 24-SMT are characterized by a basic framework constituted of a nitrogen group (protonated at physiological pH) in a lateral or cyclized chain of 5 to 8 carbon atoms, linked to a hydrophobic carrier. Efforts to mimic the structural and three-dimensional characteristics of these azasterols for purposes of rational drug design by preparing non-steroidal compounds and testing them as inhibitors of the 24-SMT have been met with mixed success [52].

## Evaluation of Novel Substrate Analogs as Mechanism-based Inactivators of 24-SMT

**Figure 6:**
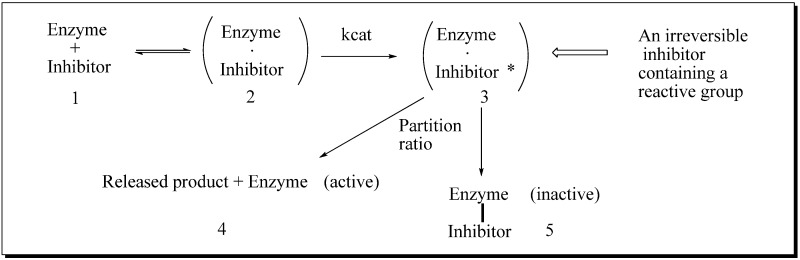
Process for mechanism-based enzyme inactivation **1** to **3** to **5**.

24-SMT inhibition proceeds by treating the enzyme with sterol-like inhibitors that bind reversibly or irreversibly at the active site. Sterol derivatives which irreversibly inhibit the enzyme can be effective drugs that can disrupt phytosterol homeostasis because catalysis of the analog can prevent the natural substrate-product turnover reaction. For mechanistic reasons, substrate analogs designed as mechanism-based inactivators of 24-SMT should form a ternary complex and convert to reactive electrophilic (or alkylating) groupings. Such reactive intermediates are active in covalent bond formation once they enter the transition state coordinate (*k*_cat_); the resulting positive charge on the intermediate can be captured by an active site nucleophile followed by enzyme inactivation that ends catalysis, [53] thereby ending the production of ergosterol or sitosterol. Kinetically, time-dependent inactivation values are determined from plots of half-time of inactivation, i.e., the time required to decrease the enzymatic activity by 50%, versus the reciprocal of the inhibitor concentration. In the case of 24-SMT, protection by the native substrate is notable at 30 µM and complete by 100 µM [11,45,47]. This suggests that analog can bind to enzyme-inhibitor complex in a competitive manner consistent with the model shown in Figure 6.

If the *k*_app_ (*k*_inact_) is slow relative to the reversible binding of inhibitor with the enzyme (*K*_i_), then the *k*_inact_ value for an inhibitor is greater than the *K*_i_ value. The greater the partitioning toward inactivation (kill) versus turnover (typical product formation), as measured in the *k*_inactivation_ term and in loss of product formation detected chemically, the more potent the inhibitor. Direct evidence for the covalent nature of binding can be established by incubation of the radiolabeled substrate with pure enzyme, then analyzing a radiofluorogram of the ligand bound protein using SDS-PAGE (polyacrylamide gel electrophoresis) [47]. The active site amino acid bound to the ligand can be determined through proteomic approaches, including mass spectrometry and radio-tracer techniques. The stoichiometry of ligand binding to the 24-SMT can be established through equilibrium binding experiments [47].

**Figure 7:**
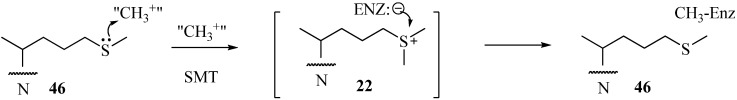
Proposed mechanism for the inhibition of C24-methylation activity by **46**.

Early analysis of the inhibition of 24-SMT by sulfur-containing analogs revealed that the uncharged thioether **46** can inhibit ergosterol biosynthesis in cultured cells at the C24-methylation stage of the pathway in similar fashion to the charged sulfonium analog **22** [11,44,46] (Table 1). However, the IC_50_ values with respect to growth inhibition and *K*_i_ values toward 24-SMT binding are different for **46** and **22** generally by one to two orders of magnitude. The kinetics for **46** and related sulfur derivatives assayed with plant or fungus 24-SMT treatment afford irreversible and time- dependent kinetics compared to the reversible and non-competitive kinetics displayed by **22** against 24-SMT. Marked differences in the growth response and enzyme kinetics to the two inhibitors have been interpreted that **46** can act as a mechanism-based inhibitor of the C24-methylation reaction (Figure 7).

**Table 1:** Examples of the effect of sterol modifications on growth inhibition.

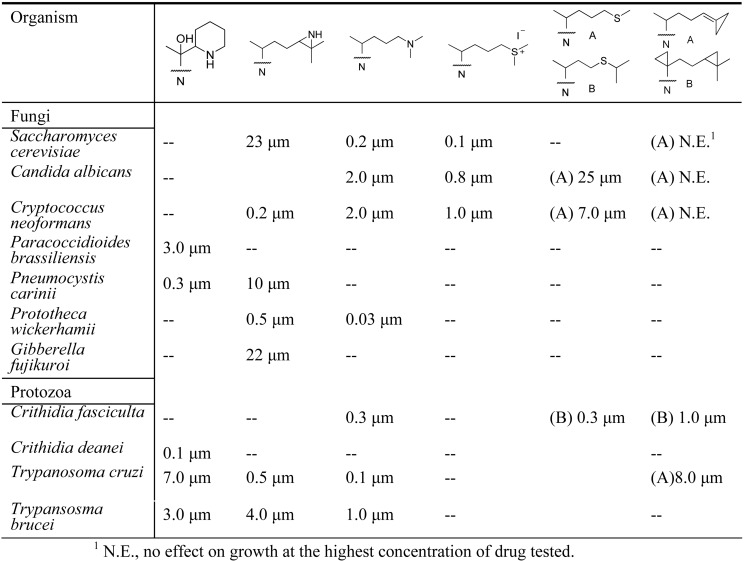

The presumption that **22** can be a substrate rather than a transition state analog of the C24-methylation reaction may result from different conformations the enzyme assumes at the outset of binding **22** and **46**, thereby sequestering the drug into distinct active site pockets that influence its mode of action [11]. Because the uncharged sulfur-based drugs are readily accumulated by pathogenic microbes yet appear to have no effect on growth or cholesterol biosynthesis in human cultured cells [54], suggest that they may be extremely effective in regulation of 24-SMT in those organisms where high substrate levels prevail due to culture conditions. In the case of sulfur-based mechanism-based inactivators tested with a fungal 24-SMT, *K*_i_ of 5 µM and *k*_inact_ values of 0.013 min^-1^ have been determined for **46** versus a *K*_m_ of 25 µM and *k*_cat_ of 0.036 min^-1^ for zymosterol **C6**, the natural Erg6p substrate. Similar observations have been reported for related sulfur analogs tested with various 24-SMTs (Table 1). 

**Figure 8:**
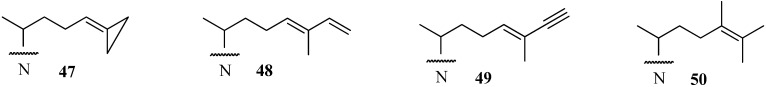
Side chain structures of substrate analogs that mimic Δ^24^-sterols acceptors.

A new and very promising generation of 24-SMT inhibitors has emerged in the last decade that offers an inactivating type of sterol side chain distinct from the one described for sulfur-containing derivatives as shown in Figure 8. These compounds exemplified by **47**, **48** and **49** are prepared synthetically [53,55,56], whereas compound **50** is a natural product that is part of the sterol C24-alkylation pathway in plants and protozoa [32,33]. In these cases, the structure of the sterol side chain is modified to afford fragmentative processing by 24-SMT to methyl product in one mode of reaction or in an alternate mode generates a highly reactive species bearing a positive charge in a region of the active site not normally occupied by the natural intermediate; this conformationally distinct high energy intermediate can be intercepted by an active site nucleophile yielding inactive enzyme. 

In our initial efforts to prepare mechanism-based inhibitors of 24-SMT, the terminal sterol side chain isopropyl group at C26/C27 was selected as the site of chemical modification because it is close to the critical double bond undergoing C24-methylation and the resulting olefin should retain the essential nucleophilicity required in catalysis. In the case of **48** and **49** methylation of the side chain occurred at C24 yielding an intermediate turned over to a 24(28)-methylene product or one that becomes bound to the 24-SMT in a covalent linkage. Compound **50,** in similar reaction to **48** and **49,** generated a C24-methylated intermediate by the soybean 24-SMT that converted to product **14**, or the intermediate was trapped by an active site nucleophile.

**Figure 9:**
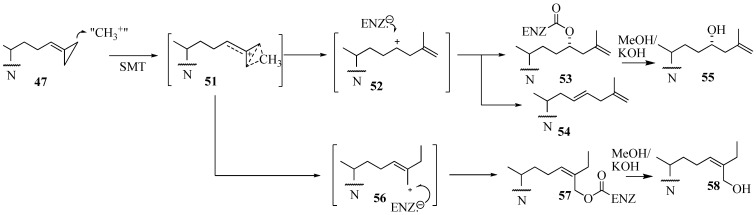
Proposed C24-alkylation pathway of 26, 27-dehydrozymosterol catalyzed by 24-SMT.

In contrast to the C24-methylation paths outlined for **48** and **49** catalysis, conversion of **47** by ScSMT followed a novel route in which the carbon atom at C26, rather than at C24, was shown to be the site of methylation. This catalysis resulted in an unprecedented C26 methylation and methylenecyclopropane ring opening generating intermediate **51** that partitions along separate paths to **52** and **56**. Trapping an active site residue by **52** has been reported to lead to enzyme inactivation [37,57]. On the other hand, **56** can also participate in covalent bond formation in the active site. The turnover product **54** and inactivation products generated by saponification of the enzyme extract **55** and **56** have been characterized by chromatographic and chemical methods to contain elongated sterol side chains. The kinetic constants for 26,27-dehydrozymosterol (DHZ) compared to zymosterol (ZY) assayed with the recombinant *Saccharomyces cerevisae* 24-SMT were as follows: DHZ, *K*_d_ 4 µM, *K*_m_ 22 µM, *k*_cat_ 0.04 min^-1^ and for ZY, *K*_d_, 4µM, *K*_m_, 17 µM, *k*_cat_ 0.65 min^-1^ [57]. Based on the product profile, we proposed the pathway shown in Figure 9. 

We surmise the mechanism-based inactivation that occurs upon incubation of 26,27-dehydrozymosterol with 24-SMT is due to irreversible, covalent binding of the inhibitor to the protein. The nature of the chemical bond (an ester bond), the chemical structures of the methyl products with methylated sterol side chains at C26 (**56** and **58**) and the amino acid(s) involved with the covalent binding (E68) have been determined for the first time for this class of substrate analog. Despite the novelty of **47**, when the compound was tested in vivo as an inhibitor of yeast growth, no inhibition was detected due to a failure of the compound to be absorbed by the cells [53,54]. On the other hand, when *Trypanosoma cruzi,* the causative agent for Chagas disease was incubated with 26,27-dehydrolanosterol, the drug was accumulated by the cells followed by a decrease in cellular ergosterol and growth inhibition, IC_50_ of 6 µM [58] (Table 1). 

Several cyclopropane derivatives have been tested with microbes with varied outcomes. When 24,25-methylene lanosterol was incubated with a sterol yeast auxotroph GL7 it was converted to 24,25-methylene ergosterol with no apparent effect on the yeast growth [54,59]. In a related study using the recombinant yeast 24-SMT assayed with 24,25-methylenezymosterol, there was no inhibition of enzyme activity at the highest concentration of analog tested of 200 µM [54,59]. However, when similar compounds were tested with a protozoan that synthesizes ergosterol, the analogs were inhibitory to growth and inhibited ergosterol synthesis at the zymosterol stage of the pathway [46] (Table 1). 

**Figure 10:**
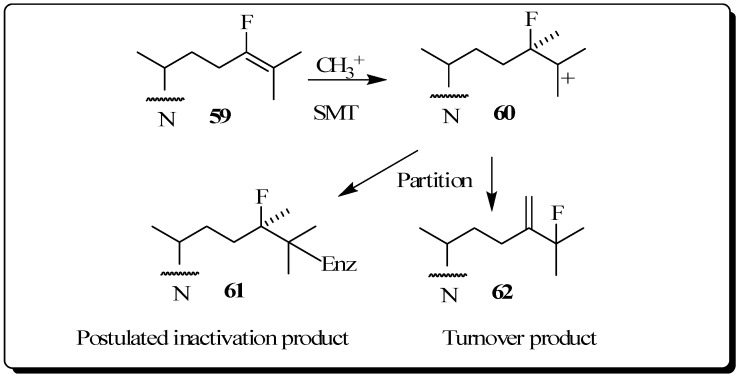
C24-methylation of 24-fluorocycloartenol by soybean 24-SMT.

The use of fluorinated substrates as mechanistic probes and inhibitors has proven to be another powerful method for obtaining information about the catalytic mechanism of various steroid transformations. The special utility of fluorinated sterol derivatives is attributed to the slight perturbation of the size and shape of the modified group on the acceptor so that the binding affinity is not affected, while at the same time the fluoro substituent exerts a strong influence on the electronic environment at the site of replacement. In order to test the effect of fluorine atom substitution on the electrophillic nature of the enzymatic C24-methylation reaction, we previously reported the kinetic, inhibition and product distribution experiments of 24-fluorocycloartenol **59** assayed with the plant 24-SMT [60]. As in the case of compounds **47** to **49**, a rate decrease of at least 10 fold for **59** compared to the catalytic rate of the natural substrate cycloartenol **2B** was found for C24-methylation, reactions considered to proceed via cationic intermediates. 24-Flurocycloartenol proved to be a potent inhibitor of plant 24-SMT (*K*_i_ = 32 µM versus *K*_m_ of cycloartenol of 30 µM) and to exhibit time-dependent enzyme kinetics (*k_inact_* of 0.32 min^-1)^, similar to that of 26,27-dehydrocycloartenol (*k*_inact_ = 0.3 min^-1^) tested with soybean 24-SMT. 24-Bromocycloartenol inhibited the plant 24-SMTs in similar competitive fashion to the 24-fluorocyloartenol treatments. However, the bromo-compound failed to generate time-dependent inhibition activities [43,60]. These studies show that vinyl fluoro analogs of the natural Δ^24^-sterol substrate for 24-SMT can offer an attractive approach to intercept a C25 cation of the bound intermediate (Figure 10). For this reason, fluorinated sterol derivatives have promise as antiparastic or antifungal agents. Studies of the inhibitory potency of fluorinated steroids toward pathogenic microbes are warranted.

## Conclusions

Since the recognition in the early 1970s that inhibition of ergosterol biosynthesis in pathogenic fungi can be used as a chemotherapeutic or agrochemical treatment, significant advances have been made in rational drug design targeted at the 24-SMT. It would appear that inhibition of 24-SMT can become an accepted modality in the treatment of 24-alkyl sterol dependent microbial growth and associated disease development. The primary action of 24-SMT inhibitors appears to be the block of sterol C24-methylation, as shown by its inhibition, which is concentration dependent. Depletion of ergosterol and related 24-alkyl sterols results in fungistatic action or in the case of select protozoa to be toxic to the cells. While many transition-state inhibitors of 24-SMT are known, none of them have gained acceptance as therapeutics since they can also inhibit the Δ^24^-reductase involved with cholesterol biosynthesis [44]. On the other hand, sterol derivatives tailored as mechanism-based inhibitors of specific fungal or protozoan pathways has merit. Studies in progress in several laboratories over the next few years will bring new and exciting structure-function information about 24-SMT and its inhibition to the public which can offer new therapy for diseases linked to ergosterol production and processing. 
